# Long-Acting Reversible Contraception and Unintended Pregnancy Among U.S. Active Duty Service Members, 2017–2018

**Published:** 2025-05-01

**Authors:** Clinton Hall, Monica Burrell, Zeina G. Khodr, Celeste J. Romano, Gia R. Gumbs, Ava Marie S. Conlin, Kelly O. Elmore

**Affiliations:** Deployment Health Research Department, Naval Health Research Center, San Diego, CA; Leidos, Inc., San Diego; Walter Reed National Military Medical Center, Bethesda, MD

Long-acting reversible contraception (LARC) includes forms of birth control that offer strong combinations of efficacy, safety, and convenience, such as subdermal implants and intrauterine devices (IUDs). ^1^ In the U.S., LARC use is estimated to be twice as prevalent among active duty service members (ADSMs) compared with the general population (23.0% vs. 10.4%). ^2 , 3^ The number of active duty LARC users increased by 19.0% between 2016 and 2019, from 50,365 to 59,942. ^2^

While unintended pregnancy following LARC placement is rare (<1.0%), ^1 , 4^ there is a paucity of research among ADSMs, a population in which unintended pregnancy has major implications for military readiness. ^5^ This descriptive study examined demographic, military, and medical characteristics of unintended pregnancies diagnosed after LARC placement in U.S. ADSMs, including pregnancies due to LARC failure as well as pregnancies undetected at time of LARC placement.

## Methods

This study utilized medical encounter data from the Military Health System Data Repository, military personnel data from Defense Manpower Data Center (DMDC), as well as data abstracted from patient electronic health records (EHRs). Medical encounter data included care received at either military or civilian facilities coded with International Classification of Diseases (ICD) and Current Procedural Terminology (CPT) codes. Data were linked using the unique Electronic Data Interchange Personal Identifier assigned to each ADSM.

The study population included confirmed cases of unintended pregnancy diagnosed after LARC placement. Suspected cases were first screened from medical encounter data; this group included ADSMs who had LARC placement at a military clinic in 2017 or 2018 (ICD-10 diagnosis code Z30.430; ICD-10 procedure codes 0UH[9,C]xHZ; CPT codes 11981, 58300), indication of pregnancy within 12 months after placement (ICD-10 diagnosis codes Z32.01, Z[33,34,36].x, O[03,09,20].x, O26.3), and no indication of LARC removal or re-insertion prior to pregnancy (ICD-10 diagnosis codes Z30.43[2,3]; ICD-10 procedure codes 0UPDxHZ; CPT codes 11976, 11982-83, 58301). Suspected cases were confirmed using information abstracted from patient EHRs; all cases had no records of LARC removal prior to pregnancy diagnosis and were described as unintended pregnancies.

Cases were categorized into 2 types, based on estimated timing of conception relative to placement: LARC failure or undetected pregnancy at placement. Date of conception was calculated by adding 2 weeks to the date of last menstrual period (LMP), as obtained from patient self-reporting or pregnancy ultrasound records. ^6^ Estimated dates of conception occurring more than 1 week after LARC placement were considered LARC failures, while all others were estimated to be already pregnant at LARC placement.

All variables were abstracted from patient EHRs except for race and ethnicity, marital status, education, rank, and service branch, which were derived from DMDC files from the month of LARC placement. Characteristics were assessed overall and by outcome type (LARC failure or undetected pregnancy at placement) using descriptive and summary statistics. Statistical analysis was completed using SAS Enterprise Guide, version 8.3.

Institutional Review Board approval for this study was obtained from the Naval Health Research Center (protocol NHRC.1999.0003) and informed consent was waived per 32 Code of Federal Regulations §219.116(d).

## Results

Initial screening identified 466 ADSMs with suspected pregnancy within 1 year following LARC placement that occurred in 2017 or 2018. After EHR review, 76 (16.3%) cases were confirmed as unintended pregnancies, of which 42 (55.3%) occurred in ADSMs who experienced LARC failure and 34 (44.7%) were among those with undetected pregnancy at LARC placement. Most cases had determined LMP from patient self-reporting at time of LARC placement versus ultrasound dating (72.4 vs. 27.6%; data not shown).

Most cases occurred among ADSMs aged 18-24 years at LARC placement (55.3%), married (52.6%), and of enlisted military rank (94.7%) **(Table 1)** . Compared with patients who experienced LARC failure, those with undetected pregnancy at placement were younger and more likely of non-Hispanic Black race or ethnicity, junior enlisted rank, in the Army, and never previously pregnant. Most LARC failures occurred among patients with an IUD (88.1%), while nearly all patients already pregnant at placement received a subdermal implant (91.2%) **(Table 2)** . Over-all, almost all cases (94.7%) had completed a pregnancy test prior to LARC placement. Almost half of cases (43.4%) ended in a non-live birth outcome.

**TABLE 1.** Demographic, Military and Clinical Characteristics of U.S. Active Duty Service Members with LARC Placement in 2017–2018 and Diagnosed Unintended Pregnancy in the Following Year, Overall and by Outcome Type

Characteristics ^a^	All Unintended Pregnancies		Outcome Type			
			LARC Failure		Undetected Pregnancy at Placement	
	No.	%	No.	%	No.	%
Total	76	—	42	—	34	—
Age, y						
18-24	42	55.3	21	50.0	21	61.8
25-34	21	27.6	12	28.6	9	26.5
>=35	13	17.1	9	21.4	4	11.8
Mean ± SD	25.2 ± 5.6		26.9 ± 6.3		23.2 ± 3.9	
Median (IQR)	23 (21 – 29)		24 (23 – 32)		23 (20 – 25)	
Race and ethnicity						
Hispanic	17	22.4	10	23.8	7	20.6
Black, non-Hispanic	25	32.9	10	23.8	15	44.1
White, non-Hispanic	24	31.6	16	38.1	8	23.5
All other groups ^b^	10	13.2	6	14.3	4	11.8
Marital status						
Married	40	52.6	24	57.1	16	47.1
Unmarried	36	47.4	18	42.9	18	52.9
Education						
Less than bachelor's degree	68	89.5	35	83.3	33	97.1
Bachelor's degree or greater	8	10.5	7	16.7	1	2.9
Military rank						
Junior enlisted (E1-E3)	29	38.2	10	23.8	19	55.9
Enlisted (E4-E9)	43	56.6	28	66.7	15	44.1
Officer	4	5.3	4	9.5	0	0.0
Service branch						
Army	29	38.2	12	28.6	17	50.0
Navy	23	30.3	19	45.2	4	11.8
Air Force	14	18.4	7	16.7	7	20.6
Marine Corps	10	13.2	4	9.5	6	17.6
Gravidity						
0	29	38.2	10	23.8	19	55.9
1+	47	61.8	32	76.2	15	44.1
Parity						
0	31	40.8	12	28.6	19	55.9
1+	45	59.2	30	71.4	15	44.1
Body mass index (kg/m ^2^ )						
<18.5	0	0.0	0	0.0	0	0.0
18.5 – 24.9	26	34.2	14	33.3	12	35.3
25.0 – 29.9	31	40.8	14	33.3	17	50.0
>=30.0	19	25.0	14	33.3	5	14.7
Patient medical history						
Cesarean section	9	11.8	7	16.7	2	5.9
Diabetes	0	0.0	0	0.0	0	0.0
Fibroids	0	0.0	0	0.0	0	0.0
Hypertension	1	1.3	1	2.4	0	0.0
Obesity	14	18.4	9	21.4	5	14.7
Pelvic inflammatory disease	2	2.6	0	0.0	2	5.9
Sexually transmitted infection	6	7.9	3	7.1	3	8.8
LARC expulsion	3	3.9	3	7.1	0	0.0

Abbreviations: LARC, long-acting reversible contraception; y, years; SD, standard deviation; IQR, interquartile range; E, Enlisted; kg, kilogram; m, meter.

a Refers to patient characteristics at time of LARC placement.

b All other race and ethnicity groups include American Indian or Alaska Native (overall n=2), Asian or Pacific Islander (n=3), those with more than 1 race or ethnicity reported (n=4), and those with missing data (n=1).

**TABLE 2.** LARC Placement and Pregnancy Characteristics Among U.S. Active Duty Service Members with LARC Placement 2017–2018 and Diagnosed Unintended Pregnancy in the Following Year, Overall and by Outcome Type

Characteristics	All Unintended Pregnancies		Outcome Type			
			LARC Failure		Undetected Pregnancy at Placement	
	No.	%	No.	%	No.	%
Total	76	—	42	—	34	—
LARC type						
Implant	36	47.4	5	11.9	31	91.2
Hormonal IUD	23	30.3	20	47.6	3	8.8
Copper IUD	17	22.4	17	40.5	0	0.0
Placement characteristics						
Pregnancy test prior to placement	72	94.7	39	92.9	33	97.1
Postpartum placement ^a^	6	7.9	5	11.9	1	2.9
Days from last menses to placement						
<=7 days	12	15.8	12	28.6	0	0.0
>7 days	64	84.2	30	71.4	34	100
Placement provider type						
Certified nurse midwife	12	15.8	7	16.7	5	14.7
Nurse practitioner	17	22.4	9	21.4	8	23.5
Physician	42	55.3	24	57.1	18	52.9
Physician assistant	3	3.9	1	2.4	2	5.9
Other or unknown	2	2.6	1	2.4	1	2.9
Months from most recent delivery to placement ^b^						
Mean ± SD	25.1 ± 36.8		23.1 ± 38.1		29.5 ± 35.0	
Median (IQR)	6 (2 – 33)		2 (2 – 25)		12 (4 – 47)	
Confirmatory imaging of LARC						
Retention	12	15.8	10	23.8	2	5.9
Expulsion	13	17.1	13	31.0	0	0.0
Perforation	1	1.3	1	2.4	0	0.0
No imaging records available	50	65.8	18	42.9	32	94.1
Months from placement to pregnancy identification						
Mean ± SD	4.5 ± 3.8		7.1 ± 3.1		1.3 ± 1.4	
Median (IQR)	4 (1 – 7)		7 (5 – 10)		1 (0 – 2)	
Pregnancy outcome						
Live birth	43	56.6	26	61.9	17	50.0
Stillbirth	1	1.3	1	2.4	0	0.0
Ectopic pregnancy	5	6.6	3	7.1	2	5.9
Spontaneous abortion	12	15.8	7	16.7	5	14.7
Elective or therapeutic abortion	15	19.7	5	11.9	10	29.4

Abbreviations: LARC, long-acting reversible contraception; IUD, intrauterine device; SD, standard deviation; IQR, interquartile range.

a Postpartum placement was defined as those occurring within 6 weeks after delivery.

b This measure included 42 service members with a known prior delivery.

## Discussion

In this study of unintended pregnancies diagnosed after LARC placement, the majority of cases occurred among young, enlisted ADSMs; about half of cases were unmarried, and one-third were never previously pregnant. These characteristics generally suggest a patient population with reduced social support and limited independence from the military, which providers should be aware of when offering LARC-related care.

The American College of Obstetricians and Gynecologists guidelines state that implants and IUDs can be placed any time during the menstrual cycle if there is reasonable certainty a patient is not pregnant. ^1^ This study found only 34 instances in which providers were unable to detect or reasonably rule out pregnancy at contraceptive initiation, suggesting some variation in recommended practice guidelines, ^1 , 7^ or incomplete or inaccurate dating of patients' recent sexual histories or LMPs, albeit rare.

To be reasonably certain a patient is not pregnant at LARC placement, providers should follow the pregnancy checklist recommended by the Centers for Disease Control and Prevention, as these criteria are highly accurate in ruling out pregnancy (i.e., negative predictive value of 99–100%). ^7^ Editing checks within EHRs (e.g., re-entering dates to facilitate patient recall and avoid entry errors) and improved patient-provider communication (e.g., through shared decision-making vs. traditional provider-driven model ^8 , 9^ ) can also help promote optimal LARC selection and timing of placement.

While calculation of a formal LARC failure rate was outside the scope of this study, only 42 failures were identified over the 2-year period, most of which were among patients with IUDs. This number is small and consistent with existing evidence that shows extremely high effectiveness (i.e., failure rates <1.0%) of LARC methods. ^1 , 4^

Study limitations include potential misclassification of outcome type (LARC failure or undetected pregnancy at placement), as date of LMP was most often derived from patient self-reporting at time of LARC placement rather than ultrasound dating. Available EHRs lacked information on how a provider ruled out pregnancy at LARC placement (other than performing a pregnancy test) or determined or corrected LMP from ultrasound records, further hindering LMP estimate reliability. Among the 466 ADSMs screened for unintended pregnancy following LARC placement, only 16.3% were confirmed cases, demonstrating the value of EHR use to identify true unintended pregnancies. Remaining cases were not confirmed due to various circumstances (e.g., no pregnancy, LARC removal before pregnancy). Limited resources did not allow for abstraction of LARC placements with no pregnancy; therefore, this study could not determine whether certain characteristics were associated with an increased risk for unintended pregnancy or LARC failure.

The consequences of unintended pregnancy can be significant for ADSMs, their support systems, and service units (e.g., extended non-deployability). If unintended pregnancy occurs while in a military theater of operation, increased costs incur due to medical evacuation and personnel replacement. Independent of deployment status, unintended pregnancy also results in quality of life adjustments for ADSMs as well as their unit personnel, with potential effects on ADSM retention, mission readiness, and unit cohesion. ^5^ Active duty status may also influence a service member's decision to maintain a pregnancy. Ongoing efforts to improve contraceptive access, use, and reliability are critical for preserving operational readiness and career advancement opportunities among this population, while also decreasing health care expenditures.
